# Binding binding: Departure points for a different version of the perceptual retouch theory

**DOI:** 10.2478/v10053-008-0013-4

**Published:** 2008-07-15

**Authors:** Talis Bachmann

**Affiliations:** Department of Psychology and Institute of Law, University of Tartu

**Keywords:** masking, consciousness, perceptual retouch, thalamic modulation, synchronization, gamma-oscillations

## Abstract

In the perceptual retouch theory, masking and related microgenetic phenomena were explained as a result of interaction between specific cortical representational systems and the non-specific sub-cortical modulation system. Masking appears as deprivation of sufficient modulation of the consciousness mechanism suffered by the target-specific signals because of the temporal delay of non-specific modulation (necessary for conscious representation), which explicates the later-coming mask information instead of the already decayed target information. The core of the model envisaged relative magnitudes of EPSPs of single cortical cells driven by target and mask signals at the moment when the nonspecific, presynaptic, excitatory input arrives from the thalamus. In the light of the current evidence about the importance of synchronised activity of specific and non-specific systems in generating consciousness, the retouch theory requires perhaps a different view. This article presents some premises for modification of the retouch theory, where instead of the cumulative presynaptic spike activities and EPSPs of single cells, the oscillatory activity in the gamma range of the participating systems is considered and shown to be consistent with the basic ideas of the retouch theory. In this conceptualisation, O-binding refers to specific encoding which is based on gamma-band synchronised oscillations in the activity of specific cortical sensory modules that represent features and objects; C-binding refers to the gamma-band oscillations in the activity of the non-specific thalamic systems, which is necessary for the O-binding based data to become consciously experienced.

## INTRODUCTION

When visual cognition is studied from an interdisciplinary perspective, researchers typically try to understand how the specific data-processing modules in the cortex mediate perception of and attention to features, objects, and events. It was only in the eighties when researchers of cognitive processes began to pay attention also to the contribution of the so-called non-specific systems of modulation to the perceptual and attentional processes (Baars, 1988; Bachmann, 1984; Crick, 1984). As one particular instance of such an approach, the theory of masking named *perceptual retouch theory* was introduced (Bachmann, 1984, 1994, 1999).

In this theory, masking and some other related phenomena (flash-lag effect, line motion illusion, attentional facilitation by local pre-cueing, perceptual latency priming) were interpreted as a consequence of certain perturbations or unusual associations of the interactive effects of processing sub-systems within a larger set of brain systems, which are considered the very mechanism of conscious experience. Basically, masking was explained as the result of relative deprivation for specific data processing (that of the target) of the service by the processes that typically perform the function of generating conscious experience for actual sensory information. In normal perception which is accompanied by conscious experience of the perceptual object, specific data (features) about that object, as represented by the driver-neurons’ cortical activity, has to be modulated by presynaptic facilitatory input from the non-specific sub-cortical systems. Without this kind of non-specific modulation, the data represented in the specific cortical modules remains pre-conscious (Bachmann, 1984, 1994; Bogen, 1995; Crick & Koch, 2003; Llinás, 2001; Magoun, 1958; Rees, Kreiman, & Koch, 2002; Schiff & Purpura, 2002). The operation of causing pre-conscious specific perceptual information to become explicit in conscious representation was termed *perceptual retouch* by Bachmann (1984, 1994).

The spatio-temporal properties of the functioning of the specific representational systems and non-specific modulation systems enabled to be put forward a masking theory which was surprisingly well consistent with quite many empirical facts from masking experiments (Bachmann, 1984, 1994). The most important of these properties are as follows: 1. Sensory stimulation evokes both specific data coding in the cortical sensory areas (SP) and a non-specific arousal-like process in the sub-cortical (especially reticular and thalamic) centers (NSP). The delay with which evoked activity reaches cortical parts of SP is substantially shorter (e.g., a few dozen ms) than the delay with which the NSP activity or a dynamic change in NSP activity, evoked through collaterals, arrives at the designated driver neurons in the same cortical SP locations. The boost of NSP-impulses that is necessary for creating an explicit representation of sufficient saliency arrives at the cortex when the SP-processes are already more or less stabilised and their activity is about to decay.

**Figure 1.**
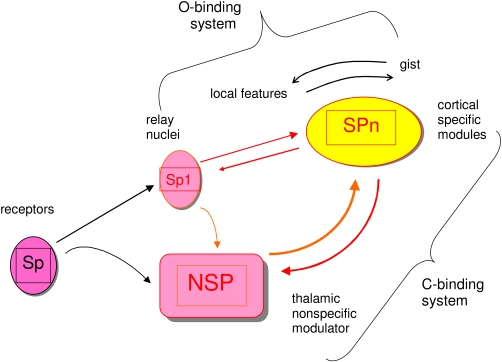
A schematic of the functional architecture of the two interacting systems for sensory data processing. Specific pathways (SP) send sensory signals upstream to the specific cortical modules that encode stimuli features and integrate objects in terms of their specific contents. This fast system builds perceptual representations also pre-consciously. A slower, non-specific system (NSP), which is located in feature-wise non-specialised thalamic and reticular centers (e.g., intralaminar nuclei, reticular nucleus, globus pallidum), interacts with cortical specific units by modulating cortical activity, preferrably in a facilitative way, increasing the frequency of firing of the specific units, decreasing their firing latency and modulating the timing of discharge patterns. The SP-system serves for binding objects from features (O-binding), the NSP system serves for modulating the activities of the O-binding system up to the level which is sufficient for explicit perception (consciousness) of the perceptual representations carried by the specific representational units. O-binding system work is necessary for the contents of conscious perception, but insufficient without the additional upgrading by the C-binding system. Both systems together are sufficient for perceptual consciousness.

2. While receptive fields of SP neurons are small and allow detailed representation, with specific contents varying from driver to driver (detector to detector), receptive fields of NSP neurons are large and unspecific regarding detailed contents (Brooks & Jung, 1973; Churchland & Sejnowski, 1992; Crick & Koch, 2003; Purpura, 1970). This property enables stimuli that are separated in space and represent different specific contents to evoke activity and interact through the activity of the same NSP unit. For instance, an initially presented stimulus (S1) evokes NSP-activity that can presynaptically modulate both the SP-units representative of S1 itself and SP-units representative of S2. These interacting stimuli need not be spatially superimposed, although they may be. (Figure 1 illustrates the functional architecture of the dual-process approach that lays the grounds for the retouch theory.)

Backward masking (including metacontrast) was explained in the following way. S1 leads to (1) fast coding within cortical SP and (2) a slower NSP-process. When S2 is presented very soon after S1 (e.g., with stimulus onset asynchrony, SOA, equal to 15 ms), a more or less simultaneous process of feature-coding and object formation is going on in SP for S1- and S2 features, and a common (“blended”) pre-conscious representation of a pseudo-object is formed. When the delayed modulation from NSP arrives presynaptically onto S1 and S2 related SP-units in the cortex, the result of retouch for consciousness will be that a blended pseudo-object is perceived. Whether both S1 and S2 can be distinctly perceived depends (a) on the intensity relations between S1 and S2 (a more intense stimulus’ features and surfaces dominating), and (b) on the spatial relations between S1 and S2 characteristics. Say, in metacontrast, where stimuli do not overlap spatially, both can be well perceived. In pattern masking with overlapping features, the perceptibility of S1 and S2 depends on the mutual camouflaging capabilities of the stimuli. Therefore, with the shortest SOAs between S1 and S2, S1 can be perceived well or not so well, depending on the peculiarities of inter-stimulus interaction within SP.

When S2 is presented after S1 with an intermediate delay (e.g., SOA = 50-80 ms), the NSP-modulation boost evoked by S1 arrives at the cortical SP at the moment when the S2 specific process is at its maximum (e.g., EPSP level is maximised), but the S1 specific process has begun to decay (e.g., EPSP level has somewhat subsided already). As a result, in the retouched perceptual image, S2 saliency is higher than S1 saliency and S2 dominates S1, as is the case in mutual masking (e.g., Bachmann & Allik, 1976; Michaels & Turvey, 1979) or in metacontrast (Breitmeyer, 1984). Subjects attend to S2 and it will replace S1 in subjective perceptual representation. With long SOAs above 150-200 ms, subjects perceive distinct successive objects – S1 and S2; both objects have had their own retouch cycles and they are entered into and held in short-term memory.

In this conceptualisation, the activity of single units was postulated to represent the activity of the whole pool of responsible neurons. Perceptual retouch theory, besides what was described above, was also able to predict perceptual latency priming (PLP, Bachmann, 1989; Neumann & Scharlau, in press; Scharlau, in press), backward masking with common-onset, asynchronous offset displays (Cohene & Bechtoldt, 1974; Di Lollo et al., 2000), a variety of psychophysiological effects where experimental facilitation of the NSP leads to unusually efficient perception of S1 (e.g., Bachmann, 1994), and some more effects. Despite this, several controversial aspects of the retouch theory became evident. While Breitmeyer and Öğmen (2000) suggested testing a unique retouch-theory prediction that there could be an illusory temporal order reversal between S1 and S2, the properties of this illusion (Bachmann et al., 2004) did not fit with retouch explanation. With PLP, the time properties of the maximum priming effect predicted by the retouch theory (at about 50-100 ms) did not conform easily to several instances of much higher PLP values found in recent experiments (e.g., Scharlau, in press; Scharlau et al., 2005).

In the retouch theory, the effects of increased visibility and saliency that ensue due to NSP-modulation were not differentially related to the contour system and surface representation system responses. However, manifold evidence shows that time-course functions of masking can substantially differ for those two perceptual properties of objects in masking (Breitmeyer et al., 2006; Breitmeyer & Öğmen, 2006; Ishikawa et al., 2006). Moreover, retouch theory is undeveloped to account for the intriguing differences between backward (metacontrast) masking, where the same local vernier targets and masks allow either strong masking or unmasking depending on whether the so-called shine-through test-and-mask combinations are used or not (e.g., Herzog, 2006). All this enforces thinking about the revision or additional development of the retouch theory.

But this is not all. In the retouch theory, the core mechanism was the mechanism for generating consciousness as it was understood until 1984. Since then, important developments have also changed the understanding of the mechanisms of conscious experience. Although the basic principle – SP has to be modulated by NSP in order to be able to explicitly communicate SP contents – has remained the same, many new characteristics of how SP and NSP interact so as to produce consciousness have become clearer (Bogen, 1995; Edelman & Tononi, 2000; Engel & Singer, 2001; Llinás & Ribary, 2001; Rees, Kreiman, & Koch, 2002; Sherman & Guillery, 1998; Singer, 1998; Steriade, 1996a, b; Steriade, Jones, & Llinás, 1990; Steriade, Jones, & McCormick, 1997; Ward, 2003). This also necessitates some revision of the perceptual retouch theory. The remaining part of the present article is devoted to outlining the premises for such a revision (or rather – development).

## PERCEPTUAL BINDING THROUGH SYNCHRONISED OSCILLATIONS

In the retouch theory there are two systems: (1) SP for stimulation content representation and (2) NSP for upgrading the selected contents of SP into consciously experienced, explicit representation. Let us first see what the SP does when fulfilling its representational function according to our current knowledge.

According to a widely accepted standpoint, perceptual representations are formed by the mutual binding of features to coherent objects (Cleeremans, 2003; Crick & Koch, 2003; Engel & Singer, 2001; Treisman, 1998; von der Malsburg, 1995). But the problem is that the same feature-codes can be part of different sets of conjugated objects. A quite likely mechanism does exist that may be flexible enough to use a limited number of features (such as “letters”) for putting together a virtually endless number of objects from combined features (such as “words and sentences”), and all the time changing the integrated sets: the neurons that represent various features, the activity of which increases and decreases in synchrony (the oscillating pattern of synchronized activity), could be the very mechanism of feature binding (Churchland & Sejnowski, 1992; Edelman & Tononi, 2000; Engel & Singer, 2001; Koch, 2004). Let me term the binding of features into objects as O-binding. (See also Figure 1.)

The best candidate for carrying out feature-binding operations through neuronal synchrony turns out to be the synchronized gamma-band activity (>40 Hz) of cortical specialized driver neurons that are tuned to specific features and characteristics of environmental stimuli (Busch et al., 2006; Doesburg et al., 2005; Engel et al., 2001; Fries et al., 2001; Melcher et al., 2005; Melcher & Vidnyanszky, 2006; Tallon-Baudry et al., 2005; Womelsdorf et al., 2006). Importantly, gamma-range synchrony seems to be also able to assist pre-conscious binding in the conditions where target stimuli remain out of awareness. Thus, the SP-function in the retouch theory can be implemented by the synchronized gamma-activity of the specific cortical neurons in the sensory areas of the brain. Although the first impulses in the sensory cortex after specific stimulation can appear already within 10-30 ms, the setting of extended synchrony takes about 50-120 ms (Busch et al., 2006; Herrmann & Mecklinger, 2001; Tallon-Baudry et al., 2005). Top-down, reentrant signaling within the cortical SP-domain appears to participate in singling out the selected set of features for object representation (Engel et al., 2001; Fries et al., 2001; Lamme, 2003). Thus, feature- and object-level representations capable of exerting pre-conscious effects can be built up by fast automatic gamma-synchronisation between specific neurons in SP. Quite probably, these processes also participate in pre-conscious priming effects (e.g., Breitmeyer et al., 2005; Elliott & Müller, 1998). Evidence points to the regularity that pre-conscious representations presume more localized synchrony, while consciousness-related representations are associated with more global neuronal synchrony (Edelman & Tononi, 2000; Haynes et al., 2005; Ward, 2003).

## ATTENTION ENHANCES GAMMA-RESPONSES

 Although gamma-synchronicity is a response given also to unattended stimuli, attention and awareness-related status tend to enhance gamma-oscillations. Thus, Summerfield et al. (2002) showed that awareness of backward-masked stimuli correlated with gamma-activity in occipital and temporal cortices. High-contrast, small, periodic stimuli elicit gain and synchrony of gamma responses in visual areas when the stimuli are attended (Womelsdorf et al., 2006). Yet, unattended stimuli also evoke a burst of gamma activity, although the spike-field coherence is smaller than in attended conditions. The onset-related firing rate was maximal at about 150 ms, post-stimulus. In a shape-tracking task, successful allocation of attention enhanced gamma-response (Taylor et al., 2005). But unattended changes in visual shapes also were accompanied by gamma boosts. Thus attention necessarily boosts gamma responses, but cannot be regarded as a sufficient mechanism for consciousness. In binocular rivalry, transient bursts of increased global phase synchrony in the gamma band were associated with visibility (Doesburg et al., 2005). As in rivaly no strong input transients are involved and because the gamma-band activity begun to peak 400-250 ms before subjects responded to the change, all this may point to the possibility that we deal here with endogeneous gamma-enhancement (an equivalent of retouch activity?) that predicts recruitment of SP-representations for consciousness. One way or another, gamma-synchrony appears to be associated with coherent conscious percepts. But again, it seems necessary, but we do not know on what conditions it also becomes sufficient. 

It is known that lateral occipital and temporal areas display gamma oscillations to attended stimuli (Tallon-Baudry et al., 2005). The latency of the response equals about 100 ms. Gamma-oscillations in the calcarine gyrus are characterised by a fast-emerging, high-frequency pattern (even more than 70 Hz). In a visual discrimination task that involves feature binding, gamma-response to an attended object emerges within only 50-150 ms (Herrmann & Mecklinger, 2001).

In the author’s present thinking, both attention and the consciousness-related property of perception are strongly associated with gamma-frequency brain activities, but the double dissociation for (1) attention-related gamma activity and (2) consciousness-related gamma activity is yet to be demonstrated in numerous replication studies. The arguments why I prefer not to put an equation mark between attention and consciousness can be found in Bachmann (2006). Most importantly, fully focused and intense attending to a stimulus or location (e.g., in metacontrast masking, binocular rivalry or motion-induced blindness) that also brings about a gamma burst in the brain does not automatically guarantee consciousness for the attended to or expected stimulus. And vice versa: for information processing that is biased and facilitated by selective attention, and that should produce gamma enhancement, there is no guarantee that the corresponding stimulus-information becomes consciously apprehended (e.g., Jaśkowski et al., 2002; Kentridge et al., 2004). Indirectly, this supports the idea that we need to have not only one variety or mechanism of gamma-activity as related to attention/consciousness, but it may be better to look for at least two brain systems prone to gamma-range dynamics when selectively processing information, but at the same time possessing relative functional autonomy. This is what fits with the agenda of the following part of this article.

## CONSCIOUSNESS APPEARS TO OPERATE THROUGH SYNCHRONISED NSP-ACTIVITY

It is well known that even unconscious brains can respond to specific sensory input in a selective and feature-wise adequate ways (de Gelder, de Haan, & Heywood, 2001; Dehaene & Naccache, 2001; Dixon, 1981; He, Cavanagh, & Intriligator, 1996; Jaśkowski et al., 2002; Kinoshita & Lupker, 2003; Marcel, 1983; Morris, Öhman, & Dolan, 1998; Moutoussis & Zeki, 2002; VanRullen & Koch, 2003), including persistent vegetative state patients (Kotchoubey, 2005). On the other hand, relatively small injuries or narrowly localised anaesthetic targeting can render subjects totally unconscious (Baars, 1997; Bogen, 1995; Newman, 1995; Steriade & McCarley, 2005). The defining picture of brain activity which accompanies conscious experience of stimuli consists in a widespread cortical oscillatory activity in the specific modular systems (O-binding of the data content representation), which is being modulated by subcortical (thalamic and reticular) oscillatory activity generated in the so-called non-specific system (Edelman & Tononi, 2000; Llinás et al., 1998; Munk et al., 1996; Singer, 1998; Steriade & McCarley, 2005). The latter can be termed binding for consciousness or *C-binding*. (See also Figure 1.) This general understanding has been predated by earlier seminal works by Bremer (1935), Bogen (1995), Hassler (1978), Jung (1958), Magoun (1958), Moruzzi and Magoun (1949), Purpura (1970), Steriade (1997, 2000) and several others.

**Figure 2.**
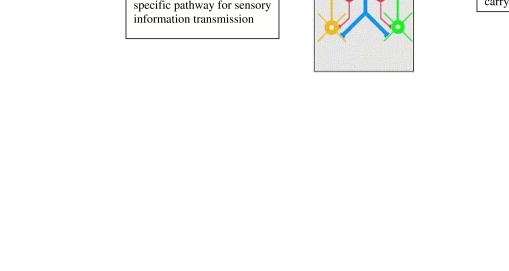
A schematic of a cortical slice where interaction between O-binding (left-side loop) and C-binding (right-side loop) systems takes place at the single-unit level. (The central part of this picture is adapted from Llinas, R.R., Urbano, F.J., Leznik, E., Ramirez, R.R., & van Marle, H.J.F. (2005). Rhythmic and dysrhythmic thalamocortical dynamics: GABA systems and the edge effect. TINS, 28(6), 325-333.) The specific pathway activates pyramidal neurons and inhibitory interneurons (upper red), producing cortical oscillations by direct activation and feedforward inhibition. Collaterals from this pathway produce thalamic feedback inhibition through the reticular nucleus (lower red). The return corticothalamic pathway (curved green arrow) from pyramidal cells returns this oscillatory loop to specific and reticular thalamic nuclei (yellow and red lower circles). The non-specific thalamocortical pathway projects to the cortex and gives collaterals to the reticular nucleus. Pyramidal neurons return the oscillation to the non-specific and reticular thalamic nuclei (green and red lower circles). This forms the second resonant loop (curved green arrow on the right). The conjunction of the specific and non-specific loops is hypothesised to generate functional binding by temporal coincidence.

One of the best models so far to describe SP/NSP oscillatory interaction in generating conscious representation has been offered by Rodolfo Llinás (e.g., Llinás, 2001; Llinás et al., 2002, 2005). A neuronal loop, including specific sensory units, contains projections onto cortical pyramidal neurons and inhibitory interneurons, and also collaterals to the NSP. A different loop includes NSP neurons located in the thalamus, which project to deeper and superficial layers of the cortex and give collaterals to the reticular nucleus and striatum and putamen. Collaterals of these two looping local circuits produce also feedback inhibition via the reticular nucleus and globus pallidus. The return pathway returns oscillations back to the reticular, specific thalamic and non-specific thalamic nuclei. When excited to respond to sensory input, both circuits produce gamma-frequency oscillations, but conscious awareness requires that these oscillations become synchronised. (See Figure 2 for an illustration of the elementary cortical module that exemplifies such an interaction.) Supralinear summation of SP- and NSP-inputs at the cortical effect layer demonstrates coincidence detection along the apical dendrites, the very mechanism of synchronised oscillatory activity. Llinás explains that coincidence detection by coactivation of SP- and NSP units provides the basis for temporal conjunction that supports cognitive binding in the conscious brain (for the details of summation and modulation see Llinás et al., 2002, 2005; coincidence detection mechanisms are well explained in detail, for instance by Börgers et al., 2005, Matell & Meck, 2004, Wang & Slotine, 2005).

Thus, oscillations that make the core of O-binding have to be associated with oscillations that make the core of C-binding, and their joined and coordinated activity is the necessary condition for a consciously experienced perceptual representation. Because the within-SP, oscillatory effect is an extended process in time (not an instantaneous “thing”), epitomising

O-binding, and because the within-NSP, oscillatory effect is also a process – C-binding for consciousness – we may indeed descibe the whole activity as “binding binding”. As long as an object is present to the senses and capable of stimulating cortical SP-neurons, O-binding represents it continually in time, but not necessarily in a conscious format unless it is supplemented by C-binding operations. As long as SP-stimulation is capable of recruiting additionally the NSP-loops’ oscillations, C-binding, by binding O-binding with itself, represents that object in conscious experience.

## VISUAL BACKWARD MASKING AND RELATED PHENOMENA IN THE LIGHT OF “BINDING BINDING”

Let me explain backward masking by the interaction of O-binding and C-binding. After having been presented, S1 evokes and sets the SP- and NSP oscillatory activity in motion. The part of modulating oscillatory activity which is caused by S1 transient becomes effective at the cortical level later than the cortical burst of SP-system gamma-oscillations for S1 had emerged. At the same later time, the gamma-burst of S2-evoked oscillatory activity is generated. C-binding has to deal with two competing oscillatory neuronal active ensembles – that for S1 (already decaying) and that for S2 (showing the most-vigorous, “fresh” pattern of oscillations with higher amplitude and perhaps with slightly better coherence characteristics). Moreover, reentrant signals within the cortical SP meet more driving input from S2 than from S1, which has been switched off already earlier. As a result, S2 features as bound by S2-related O-binding in SP become the prime contents to be bound for conscious experience by C-binding. S2-related synchronisations control what predominantly is the SP-counterpart of the joined SP + NSP oscillatory system. It may be important that phase coherence can be more easily driven by oscillations that have a higher amplitude, i.e., by the S2-related oscillatory activity.

Because the burst of oscillatory activity tends to diminish in amplitude and/or gamma-coherence (Busch et al., 2006; Steriade & McCarley, 2005; Tallon-Baudry et al., 2005), S2-related SP-oscillations always have an advantage over S1-related SP-oscillations when NSP-based modulatory oscillations become applied a bit later in time. Because the “focused arousal” response (Sheer, 1984; Singer & Gray, 1995) is very clearly expressed, but “lazy” in time, the stimuli that follow other stimuli in time are dominating in explicit perception in the experiments where fast-alternating presentation conditions are used.

Why is it that in metacontrast the first-coming target is often totally suppressed, although an interpretation of the retouch theory considered by Breitmeyer and Öğmen (2000, 2006) would predict some diminished, but yet existing residual SP-activity, and thus some diminished visibility when the delayed NSP-modulation arrives? We should not forget that in addition to the process of C-binding, visibility is determined also by interactions within the O-binding system. With some stimulus configurations, especially when the same or very close features could be in principle bound either with the target object or with the mask-object (e.g., perimeter edge of the disc and inner edge of the masking annulus), the conflict is out-ruled by an oscillatory process where the critical feature is bound to mask features instead of the target features and, in addition, local lateral-inhibitory interactions are quite strong. The

C-binding process finds a “partner oscillation” in the way of mask features’ representing activity, while the target features’ representing activity is nullified (likely out-of-phase and/or decayed). This explanation is not very good for some substitution-masking effects though.

A standard paradigm for substition masking presents a target (e.g., Landolt ring with a gap) together with the distractor stimuli (e.g., other Landolt stimuli at different spatial positions). The target is marked by a mask that consists of four dots surrounding the target. Target and mask are presented together, but when the target and distractors are switched off, the mask is the only stimulus that stays on for a variable time (a common onset, asynchronous offset method). If the target were presented alone and masked with this type of mask, there would be no masking and the target would be well visible. This is why this is sometimes called a weak mask. But if there is positional uncertainty of the target due to distractors and a larger load on attention, the same mask is effective in producing severe masking (especially with longer offset delays).

Perhaps the reason why there is no metacontrast with the so-called weak masks in substitution masking (in the trials with no distractors) has to do with the lack of conflict between target and mask features. They are not competitors within the O-binding processing activity, but are moderate competitors for the C-binding resources. This competition shows up only when distractors are present and C-binding oscillations therefore take longer to arrive at respective cortical sites. On the other hand, even when the presence of distractors help to lead to effective substitution masking of the otherwise well-visible target, masking is diminished or eliminated when spatial attention is directed to the target location before its presentation (Enns, 2004). In terms of the revised retouch theory, the pre-cue evokes C-binding processes ahead in time and when the target appears, SP-oscillations are quickly integrated into the synchronised NSP+SP, oscillatory ensemble. The target becomes visible at once.

According to the results of our recent study (Luiga & Bachmann, in press), release from substitution masking is obtainable with local spatial pre-cues, but not with central pre-cues that direct spatial attention in an abstract, encoded format (and this holds even for very long SOAs between pre-cue and target-plus-mask stimulus, where there is plenty of time for the pre-cue to be processed and interpreted). My explanation is that it is difficult to engage a sufficiently effective localised (receptive-field-centered) process of NSP-oscillations with central pre-cues; the C-binding oscillatory wave has to propagate far in cortical tissue and, consequently, (1) it takes time, (2) phase coherence suffers, (3) oscillatory amplitude decreases. As a result, central pre-cue is not effective and the target is not retouched for consciousness in a salient enough capacity. What matters is not attention (as such), but the conditions that enable evocation of a burst of coarsely localised oscillatory and facilitating activity instead.

As stated before, gamma oscillations are sensitive to input novelty and onsets. The most distinct burst of gamma activity emerges about 50-150 ms after stimulation onset. This means that when a stream of input stimuli is presented with no long empty intervals between the stream items inserted, the stimuli appearing in the epoch of the stream that covers 50-150 ms after stream onset have to benefit from the relatively more facilitated binding process. We can have subjects perform an identification task where two successive and spatially overlapping targets (S1 and S2) are presented with varying SOAs and within a stream of otherwise invariant stimuli (e.g., letter I flashed repetitively as a stream at the same position in a RSVP manner, with stream item frequency of about 20-60 Hz). And we can vary the stream epoch within which the targets that are to be identified are inserted in between the stream items. Indeed, when successive targets are presented within invariant-item streams, S1 dominates S2 in explicit perception exactly within the first stream epoch, but this pattern of relative visibility of the two targets returns to the typical S2 > S1 at later stream epochs (Bachmann & Sikka, 2005). Appearance of a stream seems to cause a burst of gamma activity, maximised (in terms of amplitude and/or coherence) at 50-150 ms post-onset, and everything that comes in at that time is facilitated. (Indirect support for this conjecture came also from a study by Bachmann and Oja, who found that the flash-lag effect, measured in terms of how much an in-stream target becomes visible faster than an isolated target, was maximised up to about 80 ms within 50-150 ms after stream onset, but reduced to about 30 ms at later stream epochs – see Bachmann, 2006.)

An intriguing set of experimental findings has been introduced by Michael Herzog’s team (e.g., Herzog, 2006). They often use small vernier stimuli as targets that have to be discriminated – whether a minute spatial displacement of an upper vertical bar away from collinearity with a lower vertical bar is in the left or right direction. Masks are various bar- and grating like stimuli that quite closely flank the targets in space, but do not overlap with them. Therefore, the paradigm is close to metacontrast masking. Thus, a vernier target can be strongly masked by a flanking localised grating, but becomes visible when the same local grating is extended much more to the periphery (the shine-though effect). The old version of the retouch theory cannot easily account for this effect: S2 has to be preferred anyway. Now I see there a possibility to understand this discrepancy. Within the O-binding system, the more extended mask object, for whatever reason (lateral-inhibitory interactions between grating elements or belonging to a different set of visual gestalts than the local mini-grating), allows parallel and mutually non-exclusive oscillatory binding processes for S1 and S2. The later-arriving oscillatory C-process absorbs both SP-oscillatory sets. My intuition is that if we would experimentally measure the exact oscillatory response to the narrowly localised grating-mask and to the spatially extended grating-mask, and compare these responses with the oscillatory response to the vernier target, then we may find either one of the two possibilities. First, a better potential for coherence or multiplicative frequency-relation between target-evoked oscillations and mask-evoked oscillations in the case of shine-through could be found. This may be a brain-process equivalent of generating good gestalts with all parts being involved and not mutually inhibited. Secondly, it may appear that in the case of shine-through conditions, the arrival of the oscillatory burst to the mask is faster or slower relative to the arrival of the oscillatory burst to the target. By virtue of this, target and mask dynamic representations are separated in time and masking interactions are prevented. These hypotheses remain to be tested.

Feature inheritance effects (e.g., Herzog, 2006; Otto et al., 2006) are another instance of new findings from more modern masking research. Sometimes, although remaining invisible itself, the masked vernier target induces an illusory perceptual appearance of the clearly visible mask features: an actually collinear vernier-like stimulus within the masking grating appears as if depicting a shift of the vernier elements, which inherit the shift characteristic of the masked vernier. This effect could be understood as misbinding within the O-binding oscillatory system (tilted or offset feature carrying neurons remaining in the pool of the synchronised set that is dominated by the mask specific signals, thus biasing what else is involved in that compound). This misbinding becomes explicated as an illusion by the C-binding system. The nice feature of this conceptualisation is that we need not worry about the non-conscious status of the masked target. The O-binding system can work pre-consciously for a big part of the specific signals and even in parallel with the O-binding set that is being integrated with C-binding activities. For instance, the tilt feature is involved in the C-bound set, but the location feature of the target is not.

The temporal dissociation of different aspects of masking, such as between contour- and brightness-processing mechanisms (Breitmeyer et al., 2006; Ishikawa et al., 2006), as well as absence of metacontrast with opposite-polarity luminous targets and masks (Becker & Anstis, 2004), are a valuable recent addition to the masking literature. In Breitmeyer et al. (2006), meta- and paracontrast was studied, and subjects had to judge the surface brightness of target discs or else discriminate the contours of target discs (with a small edge segment cut off at different locations). Targets were masked by surrounding rings as in the many earlier classic studies. It appeared that optimum SOAs for the contour task were much shorter than those for the brightness task. In paracontrast, where the mask precedes the target in time, target contrast facilitation was found (consistent with even the earlier version of the retouch theory). Ishikawa et al. (2006) varied grating-orientation and –spatial frequency of the surface of targets and masks, and they also applied a metacontrast task requiring detection of targets. They found that at short SOAs, metacontrast magnitude strongly depended on stimulus feature specificity, whereas at longer SOAs (above 40 ms), masking demonstrated strong contrast sensitivity and low stimulus feature specificity. In the earlier retouch theory version (Bachmann, 1994) it was claimed that metacontrast is unspecific to spatial-frequency properties of the stimuli. Now this remains to be revised.

The above described effects are both accountable by assuming variations in the oscillatory activity within the O-binding system. This variation can be a function of temporal properties of the brightness, surface and contour encoding sensory systems. In some instances, parallel oscillatory activity between target-related and mask-related object binding may be possible when the channels (e.g., on-system and off-system) can involve oscillatory activity in parallel, with the result emerging that C-binding explicates both the target and mask. In some other instances, as is the case with inter-contour conflict, C-binding explicates severe metacontrast with one range of timing; in the case of brightness-processing mechanisms being involved, the timing characteristics may differ.

The earlier version of the retouch theory predicted U-shaped metacontrast functions without any further oscillatory shape of the masking function as dependent on SOA (Bachmann, 1994). If we revise the understanding of interaction between the O-binding and C-binding systems so that oscillatory processes become important, we should expect that masking functions could also show some oscillatory appearance. Because the SOAs in masking studies have mostly been varied with too large steps, it is not clear whether oscillations in masking functions are a firm reality. Some first steps in showing that oscillatory masking in the gamma-range periodicity appearing in the non-mono-tonic masking functions can be found have been taken by Purushothaman, Öğmen and Bedell (2000).

Besides masking, retouch theory was used to explain several other phenomena such as flash-lag effect, Fröhlich effect, PLP and some others as well (Bachmann, 1999, 2006). In the experiments demonstrating the flash-lag effect, two types of stimulation are juxtaposed: an object that continuously changes its feature value is presented for some time, and another object that carries an invariant feature value is briefly flashed alongside the changing object (e.g., the spatial location of a moving bar is changing or the colour of a stationary disc gradually changes from yellow to red while another bar is flashed at a stationary location as aligned with the moving bar or another disc is flashed nearby and has the same colour as the changing disc precisely at the moment of flash presentation). Flash-lag effect means an illusion where the feature value of the flashed object (e.g., location, colour) lags behind the perceived feature value of the changing object. In the Fröhlich efect (Fröhlich, 1923), the perceived first position of a moving object that comes from behind an occluder is located not at the position it actually became exposed (at the edge of the occluder), but at a position shifted forwards from the edge. In PLP, the subjective moment in time when the target object becomes visible is speeded up (visual latency decreased), provided that a priming stimulus – no matter whether it is masked to invisibility by the target or remains visible – is presented ahead in time (for about 30-100 ms).

Perceptual retouch theory has a common explanation for all these listed phenomena. The delayed NSP-modulation arrives when the SP-contents that are encoded cortically are already changed, and conscious representation includes the new feature values; it performs this build-up of conscious representation faster than it does in the case of a single stimulus presentation because the NSP-process was set in motion by the preceding stimulation. However, with PLP there seem to exist some controversies between data on the one hand and retouch theory predictions on the other hand (e.g., Scharlau, Ansorge, & Horstmann, 2005). First, as most of the robust PLP effects have been obtained by the metacontrast-like stimulation conditions (mask perception being facilitated by the preceding target), and since metacontrast interaction is a spatially very precise one (assuming small receptive fields of the critical feature representing units), the retouch explanation can be put in doubt. This is because in the original version of the theory the NSP/modulatory neurons are assumed to have large receptive fields, but PLP effects can be spatially very precise. This problem can be overcome if we understand that C-binding results depend also on the accompanying O-binding results: what is explicated for consciousness and *how* (fast) it is explicated depends also on the nature of interactions within the SP-system. Although C-binding neurons have large receptive fields and their oscillation is widespread, because O-binding neurons have small receptive fields and oscillations are more localised, the facilitating effect can be quite precise in space.

The same argument applies to the criticism suggesting that perceptual retouch as an automatic process is not open to top-down influences. For example, Scharlau et al. (2005) found that the values of the PLP depend on the judgment method for temporal order of a prime and a target. Changes come in depending on whether subjects attend to the prime or the target. But the controversy may not be fully founded because even if part of the C-binding oscillations is mostly fed-in in a feedforward manner (especially its initial burst), the O-binding processes include reentrant signalling and attentional pretuning can have its (localised and bias-related) effect. But the results of this effect have to be retouched for consciousness by the C-binding nevertheless, and the timing of visibility will ultimately depend on the latency of NSP-oscillatory application.

**Figure 3.**
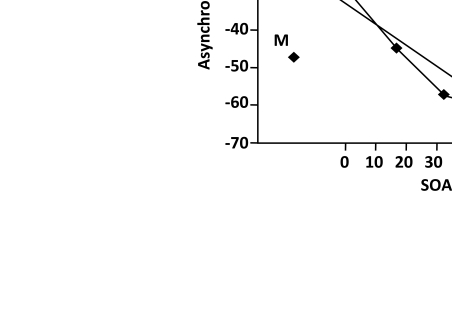
An illustration of the functional relationship relating SOA (set between prime and target) with perceptual asynchrony between targets presented in control conditions without prime and main experimental conditions where prime precedes target. The slope of the function is about 0.5. (Adapted from Aschersleben and Bachmann, 2004, unpublished.)

The intriguing feature of the PLP effect is that there is no direct correspondence between the prime-to-mask SOA and the temporal value of latency shortening due to priming (with the coefficient equal to about 0.5). If we have SOA between prime and target as the argument and the psychophysically estimated PLP value as the ordinate (see Figure 3), the old version of the retouch theory was supposed to predict PLP = SOA.

Actually, as seen in Figure 3, PLP values tend to deviate from the theoretically expected y = x, function. (Instead, y = kx seems to happen, with k equal to about 0.5.) The revised retouch theory can be specified so as to be able to explain this puzzle. We can assume that it is not the latency with which the first discharges in the cortex, caused by subcortical presynaptic NSP-facilitation, emerge that causes retouch up to consciousness. Instead, a certain critical duration of the combined oscillatory activity that is necessary for explicit representation is what matters (compare also Benjamin Libet’s and Christof Koch’s notion about a minimum duration of activity necessary for consciousness – Koch, 2004). If so, there are many possibilities to explain the 0.5 ratio between PLP values and SOA values. Term it “C-recruitment, temporal coefficient”, if you wish.

The standing wave of invisibility, metacontrast masking (e.g., Macknick & Martinez-Conde, 2004), is another new development in masking literature that needs a commentary based on the retouch theory assumptions. Usually masking is demonstrated by flashing two brief successive stimuli – the target and the mask, or vice versa. Both stimulation and the effects it brings about are so fast and short lived that it may not be very easy to make precise measurements of the effects. It is especially frustrating, considering that many modern methods of brain imaging such as fMRI or PET recquire longer state variables in order to produce good and reliable results. In the standing wave of invisibility illusion, target and mask, for instance a solid disc and a ring that snugly embraces the target, are alternatingly and continuously flashed for an extended time. With optimal temporal and luminance related parameters, it is possible to render the target effectively invisible for extended time periods spanning up to many seconds. From the revised retouch theory point of view, the effect is interpreted as both inhibitory interactions within SP where O-binding chooses the annulus instead of the disc (or flankers instead of the flanked target), and predominance of mask-related SP-oscillations in specific data binding with NSP-oscillations for the consciously experienced representation. Robust dichoptic effects of masking and weak interocular suppression between binocular neurons at the early levels of the visual cortex (op. cit.) suggest that widespread NSP-oscillations for C-binding that are interacting with SP-oscillations for O-binding are especially important when taking place in advanced visual (e.g., lateral occipital) and temporal cortical locations.

## WELL-KNOWN MASKING THEORIES AND “BINDING BINDING”

As a dual-process theory, the revised retouch theory should not be understood as an approach that is exclusive with regard to other theories. First of all, the inhibitory and misbinding interactions within the

O-binding system, which form the contents of perceptual representation that are completed for the moment of C-binding application, can be explained and have to be explained by the more specialised sensory-aspect, masking theories. Thereby, an important task is to differentiate in what circumstances masking effects directly originate from the SP/NSP interaction and the corresponding two-system actions’ relative timing dynamics, and in what circumstances retouch simply explicates the results of masking-interactions that take place within the SP-system. Related to this, we have to understand and show what the experimental conditions and stimulation properties are where the retouch theory provides a direct mechanistic explanation for the masking effects at hand, and where the very mechanism(s) of masking are independent of NSP-action (the latter simply explicates the results of masking-interaction for visual awareness).

1. The RECOD model of masking (Breitmeyer & Öğmen, 2006), which outsprung from the earlier very influential transient-on-sustained and sustained-on-sustained theory, relates to the retouch acount in the following way. The feature binding and sensory (lateral) inhibition aspects are dealt with within the set of processes of O-binding, with a special emphasis on the contour processing mechanisms. The same applies to unconscious priming effects. Saliency of surfaces (in the context of masking) and appearance of integrated, holistic objects in awareness requires involvement of C-binding processes. An interesting possibility should be to see whether, and if yes then how, the transient system action participates in the evocation of the crucial first burst of gamma-oscillations – both within SP and within NSP. The fine-tuning of the understanding of contour versus surface and contrast mechanisms’ roles in the light of C-binding mechanism’s action is also one of the prime tasks.

2. When introducing substitution masking theory, Di Lollo, Enns and their associates (e.g., Di Lollo et al., 2000; Enns, 2004) advanced some earlier accounts of attention-dependent masking effects (e.g., Bachmann & Allik, 1976; Di Lollo et al., 1974; Eriksen & Collins, 1969; Michaels & Turvey, 1979; Ramachandran & Cobb, 1995; Tremblay-Shelley & Mack, 1999) and provided a strong paradigmatic case for attention-dependent masking. From the retouch theory point of view, substitution-masking can be seen primarily as the result of delayed involvement of NSP-based C-binding oscillations after the SP-based O-binding operations (including reentrant signalling and partial decay of S1 at the pattern level in favour of S2 representation) have been already carried out. When, due to distractors, attention is dispersed, NSP-resources cannot be rigorously and rapidly invoked and mask information becomes the dominating data for retouch because C-binding becomes effective only at the moment when the O-binding process emphasises mask-object representation. When C-binding has been set on in advance, substitution masking obviously disappears, but the pre-cue has to be sensory in nature and spatially localised close to the target (Luiga & Bachmann, in press).

## ENDCOMMENTS

To end the acquaintance-tour of this sketch of the modified perceptual retouch theory, a few general remarks are necessary. Due to its emphasis on the temporally extended process of SP/NSP interaction, retouch theory naturally fits with the notions about *minimum excitatory duration*, which is necessary for a conscious percept to emerge (e.g., Libet’s or Koch’s works – see Koch, 2004), and about the importance of considering the object updating operations in addition to dealing with simple delays of first manifestations of neural (cognitive) responses after stimuli onset [e.g., Enns, Lleras, & Di Lollo’s (2006), Kahneman & Treisman’s (1984), Kanwisher’s (2001), Koch’s (2004), Neumann’s, Müsseler’s and Scharlau’s (see Scharlau, 2004) works]. The rigid onset-onset scrutiny may not be enough for understanding masking and related phenomena. Masking as the process of preventing the target from becoming consciously experienced should be analysed by temporally extended cyclic processes insofar as the very phenomena of visual awareness are based on temporally extended oscillatory processes.

The amended retouch theory appears to help build bridges between various research paradigms such as masking, flash-lag, PLP, Fröhlich effect, masked priming, pre-conscious processing, visual spatial attentional pre-cueing, and line-motion illusion, but maybe also crowding effects, motion-induced blindness, binocular rivalry, change blindness, repetition blindness and attentional blink. But this agenda remains out of the scope of the present article. In the domain of masking, the core predictor of masking strength should be the empirically tested establishment of SP/NSP oscillatory synchrony – its emergence, dynamics and maintenance in time.

## Acknowledgements

The contents of this article have benefitted from support from the Estonian Science Foundation grant #5778 and from extended discussions and “researchtogether” with the following members of my lab – Endel Põder, Iiris Luiga, Karita Hommuk, Pilleriin Sikka.
